# Definitive radiotherapy consisting of whole pelvic radiotherapy with no central shielding and CT-based intracavitary brachytherapy for cervical cancer: feasibility, toxicity, and oncologic outcomes in Japanese patients

**DOI:** 10.1007/s10147-020-01736-4

**Published:** 2020-08-27

**Authors:** Takeaki Kusada, Takafumi Toita, Takuro Ariga, Wataru Kudaka, Hitoshi Maemoto, Wataru Makino, Kazuki Ishikawa, Joichi Heianna, Yutaka Nagai, Yoichi Aoki, Sadayuki Murayama

**Affiliations:** 1 Department of Radiology, Graduate School of Medical Science, University of the Ryukyus, 207 Uehara, Nishihara, Okinawa 903-0215 Japan; 2 Radiation Therapy Center, Okinawa Chubu Hospital, 281 Miyazato, Uruma, Okinawa 904-2293 Japan; 3 Department of Obstetrics and Gynecology, Graduate School of Medical Science, University of the Ryukyus, 207 Uehara, Nishihara, Okinawa 903-0215 Japan; 4 Department of Obstetrics and Gynecology, Nanbu Medical Center/Nanbu Child Medical Center, 118-1 Arakawa, Shimajiri, Okinawa 901-1193 Japan

**Keywords:** Cervix neoplasms, Radiotherapy, Brachytherapy

## Abstract

**Background:**

This prospective study investigated the feasibility, toxicity, and oncologic outcomes of definitive radiotherapy (RT) consisting of whole pelvic radiotherapy with no central shielding (noCS-WPRT) and CT-based intracavitary brachytherapy (ICBT) in Japanese patients with cervical cancer.

**Methods:**

Patients with cervical cancer of FIGO stages IB1–IVA were eligible. The treatment protocol consisted of noCS-WPRT of 45 Gy in 25 fractions and CT-based high dose-rate ICBT of 15 or 20 Gy in 3 or 4 fractions prescribed at point A. The prescribed ICBT dose was decreased if the manual dwell time/position optimization failed to meet organs-at-risk constraints. Graphical optimization and additional interstitial needles were not applied.

**Results:**

We enrolled 40 patients. FIGO stages were IB1: 11, IB2: 13, IIA2: 1, IIB: 11, IIIB: 3, and IVA: 1. Median (range) pretreatment tumor diameter was 47 (14–81) mm. Point A doses were decreased in 19 of 153 ICBT sessions (12%). The median follow-up duration was 33 months. The 2-year rates of pelvic control, local control (LC), and progression-free survival were 83%, 85%, and 75%, respectively. Pre-ICBT tumor diameter, high-risk clinical target volume (HR-CTV), total HR-CTV D90, and overall treatment time (OTT) significantly affected LC. Late adverse events (grade ≥ 3) were observed in 3 patients (2 in the bladder, 1 in the rectum).

**Conclusions:**

Definitive RT consisting of noCS-WPRT and CT-based ICBT was feasible for Japanese patients with cervical cancer. To further improve LC, additional interstitial needles for patients with a large HR-CTV and shorter OTT should be considered.

## Introduction

Definitive radiotherapy (RT) and/or concurrent chemoradiotherapy (CCRT) consisting of external beam radiation therapy (EBRT) and intracavitary brachytherapy (ICBT) is the standard treatment of choice for patients with stage IB to IVA uterine cervical cancer [1].

In clinical practice in Japan, central shielding (CS) of 3–4 cm width has been utilized for the later part of EBRT to reduce the doses delivered to organs at risk (OARs), such as the rectum and bladder [2–4]. With this treatment strategy, favorable oncologic outcomes as well as acceptable incidence and/or grade of late toxicities have been reported from Japan [3, 5–7]. However, in the era of three-dimensional (3D) EBRT planning, the uncertainty of CS in evaluating doses to both the cervical tumor and the surrounding OARs [8, 9] has become a serious flaw. Practically, the dose from EBRT with CS (CS-EBRT) has been completely omitted (as 0 Gy) in the calculation of the total dose of EBRT and ICBT to the cervical tumor. Cumulative doses of EBRT and ICBT (i.e. high-risk clinical target volume [HR-CTV] D90, point A dose) have been reported with that method, both in actual clinical practice and in clinical studies in Japan [3, 4, 6, 7, 10–14]. Tamaki and colleagues claimed that doses from CS-EBRT should not be completely omitted from the calculation, because certain doses from CS-EBRT also contribute to the cervical tumors [15]. Some investigators have tried to develop novel methods of quantitative summation of doses from CS-EBRT with deformable image registration (DIR) [16, 17]. However, appropriate methods and clinical values have not been established.

Recently, clinical use of three-dimensional image-guided brachytherapy (3D-IGBT) has been increasing [18, 19]. 3D-IGBT enables evaluation of the dose–response relationship with the use of dose–volume histogram (DVH) parameters. In such situations, application of whole pelvic EBRT with no CS (noCS-WPRT)**,** which does not hinder the quantitative dose evaluation, would be strongly expected also in Japan.

Doses delivered to the OARs, especially for the rectum and bladder, could increase without the use of CS compared to the previous treatment with CS. Several studies of 3D-IGBT demonstrated dose–response relationships between the incidence of late complications and doses [20–22]. Therefore, we considered that the safety as well as efficacy of this approach should be evaluated in a prospective clinical study with a protocol adopting 3D-IGBT.

Based on this information, we conducted a single-institutional prospective study of definitive RT/CCRT that consisted of noCS-WPRT and CT-based ICBT to assess its feasibility, toxicity, and oncologic outcomes for Japanese patients with uterine cervical cancer.

## Patients and methods

### Study design

This was a single-institutional prospective study carried out at the University of Ryukyus Hospital and was approved by that institutional review board. All patients provided written informed consent before study entry.

### Patients

Patients with histologically proven squamous cell carcinoma, adenocarcinoma, or adenosquamous carcinoma of the uterine cervix with International Federation of Gynecology and Obstetrics (FIGO) stage IB1, IB2, IIA1, IIA2, IIB, IIIA, IIIB, or IVA were eligible. Cervical tumor diameter was assessed by MRI T2WI. Patients with paraaortic and/or common iliac lymphadenopathy (≥ 10 mm) assessed by computed tomography (CT) were ineligible. Eligibility criteria also included age 20–85 years and Eastern Cooperative Oncology Group (ECOG) performance status (PS) 0–3. There were no criteria regarding the use of chemotherapy. Patients with prior treatments (RT or surgery) to their abdomen or pelvis for cervical cancer and/or other malignancies were ineligible.

### Radiotherapy

RT consisted of noCS-WPRT and high-dose-rate ICBT.

noCS-WPRT was performed with a three-dimensional (3D) conformal 4-field box technique with 10 MV photon (Clinac iX, Varian Medical Systems, California, USA). Clinical target volumes (CTVs) were contoured according to previously published guidelines [23, 24]. noCS-WPRT was delivered 5 days a week to achieve a total dose of 45 Gy in 25 fractions. Boost EBRT of 6–10 Gy in 3–5 fractions was indicated for patients with nodular parametrial invasion to the pelvic walls, and/or internal and external iliac nodal metastases (≥ 10 mm in shortest diameter).

ICBT was administered once a week with a microSelectron HDR brachytherapy afterloader (Elekta, Stockholm, Sweden) with a standard applicator set of tandem and ovoids. A vaginal cylinder was used for patients with a narrow vagina or vaginal involvement over 1/2. Additional application of interstitial needles was not indicated. After insertion, CT images were acquired (≤ 2.5-mm slices), and treatment planning was performed for every ICBT session. To decrease doses delivered to the small bowel, the bladder could be filled with saline (approximately 100–150 cm^3^). Treatment planning was performed with Oncentra Brachy (Elekta). OARs were contoured for every ICBT session, but HR-CTV delineation was dispensable. The ICBT dose was prescribed at Point A with standard loading of the source dwell positions and weighting according to the Manchester System.

Table 1 shows the planned RT doses of the protocol. The first ICBT was performed immediately after administration of 30.6 Gy of noCS-WPRT for patients with stage IB1/IIA1 squamous cell carcinoma whose tumor diameter was less than 20 mm. Other patients started ICBT immediately after receiving 39.6 Gy of noCS-WPRT. ICBT was omitted on the day EBRT was delivered. Table 2 shows the planning aims for doses for the OARs. The doses for the rectum, sigmoid colon, and small bowel were determined based on data from previously reported series [21, 22]. Since there were no clear data on bladder dose constraint, 100% of the prescription dose at point A was employed as a planning aim for bladder. To meet these OAR dose aims, first, source dwell times were modified manually while keeping the prescribed point A dose. Graphical optimization was not allowed. In cases that failed to achieve the aims through the process, the point A dose was decreased regardless of dose coverage for the cervical tumor visualized on the planning CT images. The overall treatment time (OTT) of the RT was to be less than 56 days.

**Table 1:** Planned doses

	noCS-EBRT	ICBT^a^	Total EQD2
IB1/IIA2 SCC (< 20 mm)	45 Gy/25 Fr	15 Gy/3 Fr	63 Gy
Others	45 Gy/25 Fr	20 Gy/4 Fr	69 Gy

*ICBT* intracavitary brachytherapy, *EQD2* equivalent dose in 2 Gy per fraction (*α*/*β* = 10), *Fr* fractions, *SCC* squamous cell carcinoma

^a^Prescribed at point A

**Table 2:** Planning aims for doses of organs at risk

OAR(s)	D2 cm^3^	
	ICBT (per fraction)	noCS-WPRT + ICBT^a^
Bladder	5 Gy	75 Gy
Rectum, sigmoid colon, small bowel	4 Gy	66 Gy

*OAR* organ at risk, *ICBT* intracavitary brachytherapy, *noCS-WPRT* whole pelvic external beam radiation therapy with no central shielding

^a^noCS-WPRT + ICBT was calculated in equivalent dose in 2 Gy per fraction (EQD2) (*α*/*β* = 3)

### Follow-up

Patients were followed up every 3 months for the first 2 years. Follow-up included a pelvic examination with PAP smear. CT examination of the chest, abdomen, and pelvis was performed every 6 months. Time to progression was defined as the time from the day of noCS-WPRT start to disease progression assessed clinically or radiologically. Patients with persistent local (cervical) disease were assessed as having local recurrence with time to progression 0. Toxicity was evaluated using the Common Terminology Criteria for Adverse Events, version 4.0.

### Statistics

The primary endpoint was the 2-year pelvic control rate (2y-PC). The secondary endpoints included compliance with RT dose constraints, the 2-year local control (LC) rate, and the 2-year complication rates. The cumulative outcomes and late complication curves were estimated with the Kaplan–Meier method (SPSS version 22, IBM, New York, USA). Differences in outcomes were compared with a log-rank test. The study target enrollment was 40 patients.

## Results

### Patients

Forty patients were enrolled in the study between April 2014 and August 2016. Table 3 shows their demographic and treatment characteristics. Concurrent chemoradiotherapy with weekly cisplatin of 40 mg/ m^2^ was indicated for 29 patients with stage IB1/IIA1 cervical cancer with a tumor diameter of ≥ 25 mm or stage IIB2/IIA2/III/IVA disease. Eleven patients with small tumor diameter (less than 25 mm) and/or ages over 70 were treated with radiotherapy alone.

**Table 3:** Patient demographic and treatment characteristics

Factor	
Median age (range)	56 (34–84) years
Median BMI (range)	27 (17–42) kg/m^2^
FIGO stage (2008)	
IB1	11
IB2	13
IIA2	1
IIB	11
IIIB	3
IVA	1
Pathology	
Squamous cell carcinoma	37
Adenocarcinoma	2
Adenosquamous carcinoma	1
Median pre-treatment tumor diameter^a^ (range)	47 (14–81) mm
Median pre-ICBT tumor diameter^a^ (range)	29 (10–68) mm
Pelvic lymph node metastases^b^	
Yes/no	14/26
Median overall treatment time (range)	55 (45–68) days
Concurrent chemoradiotherapy	
Yes/no	29/11
Boost EBRT to pelvic nodes	
Yes/no	17/23^c^

*BMI* body mass index, *ICBT* intracavitary brachytherapy, *EBRT* external beam radiation therapy

^a^Assessed by MRI T2WI

^b^Lymph nodes ≥ 10 mm in the largest diameter assessed by CT/MRI

^c^3 patients received boost EBRT despite having nodes that were negative (< 10 mm)

### Feasibility

The median (range) OTT was 55 (46–68) days. The OTTs of 12 patients (30%) were over 57 days. Six of those 12 patients had unexpected RT treatment interruptions due to events including treatment-related complications and national holidays. Thirty-nine patients completed the treatment protocol. One patient failed to receive ICBT due to a poor response to prior noCS-WPRT. She received boost EBRT 10 Gy in 5 fractions to the cervical tumor.

ICBT was performed for 153 sessions in 39 patients. Tandem and ovoids were used in 38 patients, and a vaginal cylinder applicator with tandem was used in 1 patient. CT/MRI compatible applicators were used in 118 sessions, and metallic applicators were used in 35 sessions. ICBT was performed with the prescribed point A dose of 5 Gy in 131 sessions. Of these, 79 sessions were delivered without any optimization, and the remaining 52 sessions were done with dwell weight/time optimization. Eighteen sessions (12%) required reduction of the prescribed point A dose. Reduced doses were 4.5 Gy in 6 sessions, 4 Gy in 9 sessions, and 3.5 Gy in 3 sessions. Protocol deviation of the prescribed dose (6 Gy) occurred in one session. The dose difference was adjusted by decreasing the prescribed dose (4 Gy) of the next ICBT.

### Dose–volume histogram parameters (Table 4)

The minimum dose to 90% of the HR-CTV (HR-CTV D90) and the minimum dose to the maximum exposed 2 cm^3^ volume (D2 cm^3^) of the OARs were calculated in all 153 ICBT sessions. The total dose in each parameter was calculated by simply adding the dose from EBRT (the prescription dose to the cervix) and dose from ICBTs as the equivalent dose in 2 Gy fraction (EQD2).

**Table 4:** DVH parameters and feasibility according to HR-CTV

	All (*n* = 39^a^)	HR-CTV < 40 cm^3^ (*n* = 22)	HR-CTV ≥ 40 cm^3^ (*n* = 17)
HR-CTV D90	73 (60–87) Gy	75 (67–87) Gy	69 (63–74) Gy
Bladder D2 cm^3^	74 (58–95) Gy	78 (60–95) Gy	70 (62–90) Gy
> 75 Gy^b^	45% (18/40)	55% (12/22)	35% (6/17)
Rectum D2 cm^3^	57 (51–73) Gy	55 (51–63) Gy	57 (53–73) Gy
> 66 Gy^b^	8% (3/40)	0% (0/22)	18% (3/17)
Sigmoid D2 cm^3^	62 (50–71) Gy	62 (50–69) Gy	62 (54–71) Gy
> 66 Gy^b^	20% (8/40)	23% (5/22)	18% (3/17)
Small bowel D2 cm^3^	56 (45–72) Gy	57 (46–72) Gy	53 (45–67) Gy
> 66 Gy^b^	13% (5/40)	18% (4/22)	6% (1/17)

DVH parameters: total EQD2 of EBRT and all ICBTs (HR-CTV; *α*/*β* = 10, OARs; *α*/*β* = 3). HR-CTV: volume at the 1st ICBT

^a^1 patient without ICBT was excluded

^b^Target dose

Planning aims for doses of the OARs for single ICBT were achieved in 139/153 (91%) sessions for the rectum, 117/153 (76%) sessions for the sigmoid colon, 129/153 (84%) sessions for the small bowel, and 81/153 (53%) sessions for the bladder. Although most patients maintained their aims for the rectum, sigmoid colon, and small bowel, nearly half of the patients did not achieve the aim for the bladder.

At the time of the analyses, the HR-CTV was contoured (by KT and TT) according to the JROSG guidelines [^25^]. Median HR-CTV on the 1st ICBT was 35 cm^3^ (17–85 cm^3^). Among patients with HR-CTV ≥ 40 cm^3^, only 2 of 17 (12%) had point A dose reduction. In contrast, 12 of 22 patients (55%) with HR-CTV < 40 cm^3^ had point A dose reduction.

### Treatment results

**Fig. 1:**
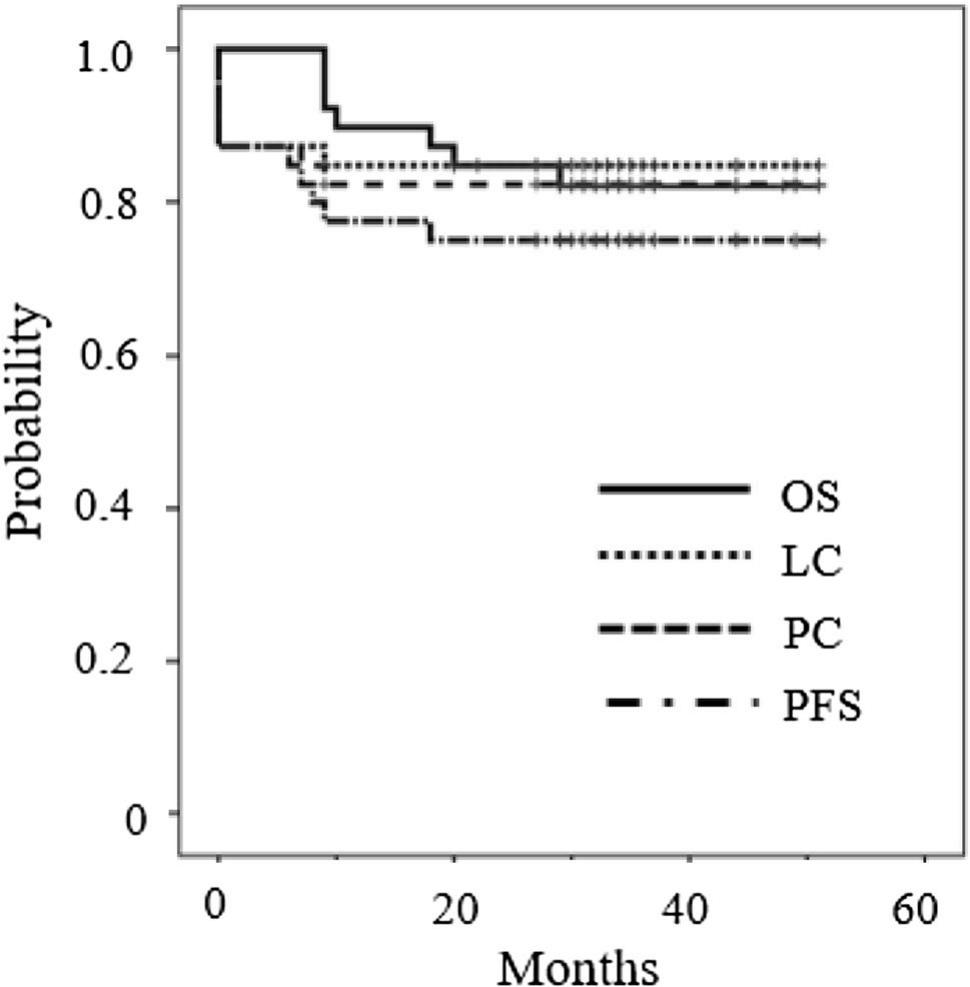
Pelvic control (PC), local control (LC), overall survival (OS), and progression-free survival (PFS) of all 40 patients

The median (range) duration of follow-up for all 40 patients was 33 (9–52) months. One patient was lost to follow-up after 22 months. Seven patients died. Of these, six died from cervical cancer, and one without cervical cancer recurrence died from an unrelated cause (sepsis). Ten of the 40 patients (25%) developed recurrence. The first sites of recurrence were as follows: cervix in 5, cervix and pelvic node in 1, pelvic node and para-aortic nodes (PAN) in 1, and distant metastases in 3 (PAN: 1, lung/liver/bone: 1, peritoneum: 1). Figure 1 shows the oncologic outcomes for all 40 patients enrolled. The 2-year pelvic control rate (PC), local control (LC) rate, progression-free survival (PFS) rate, and overall survival (OS) rate were 83, 85, 85, and 75%, respectively.

**Fig. 2:**
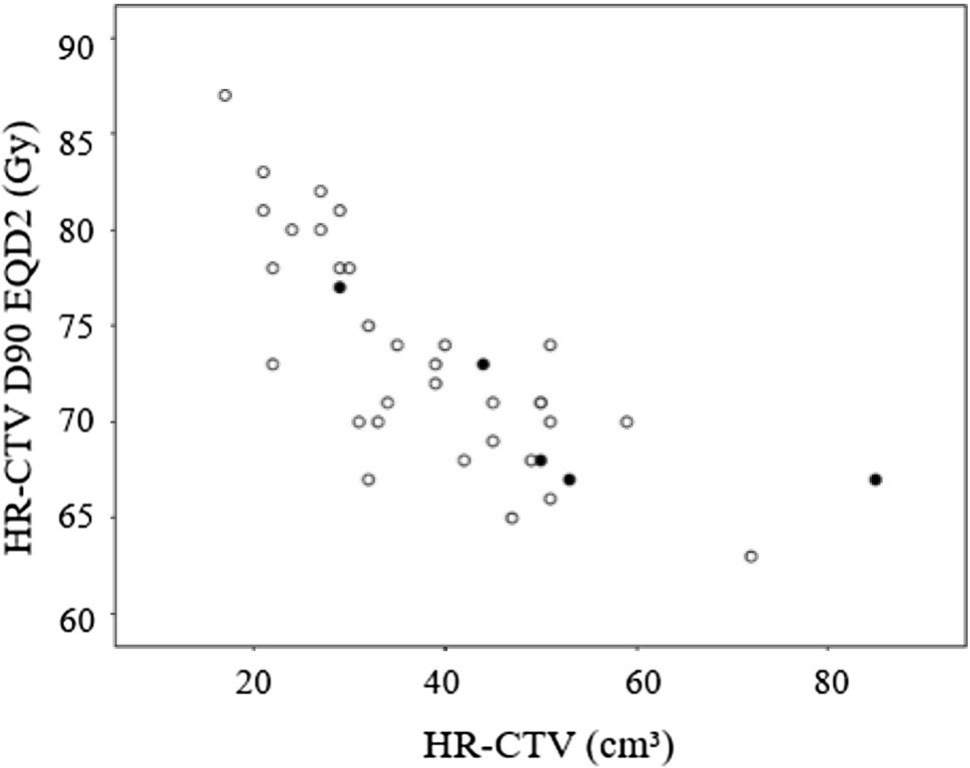
Distribution of local recurrence as a function of HR-CTV and total dose (D90) in 39 patients treated with definitive RT consisting of noCS-WPRT and ICBT. Open circles indicate patients without local recurrence. Patients with local recurrence are indicated with filled circles

Table 5 shows the 2-year oncologic outcomes according to clinical and treatment factors. Patients with large pre-treatment tumor size (≥ 40 cm^3^) appeared to have poorer LC than those with small pre-treatment tumor size (< 40 cm^3^), but the difference was not significant. Patients with pre-ICBT tumor diameter ≥ 40 mm had significantly inferior LC compared with those with pre-ICBT tumor diameter < 40 mm. Patients with HR-CTV ≥ 40 cm^3^ had worse LC than those with HR-CTV < 40 cm^3^ with marginal significance. Total HR-CTV D90 had a significant impact on LC, with a cut-off value of 70 Gy. Only 2 patients who received ≥ 70 Gy of HR-CTV D90 developed local recurrence. One patient had a pathology of adenocarcinoma, and another experienced a 14 days treatment interruption due to intracranial hemorrhage (OTT, 68 days). Figure 2 is a scatter plot that shows a significant negative correlation between HR-CTV D90 and HR-CTV (Pearson correlation coefficient – 0.745, bilateral *p* value < 0.001).

**Table 5:** Oncologic outcomes according to clinical and treatment factors

Factor	*n*	2-Year PC (%)	*p*	2-Year LC (%)	*p*	2-Year PFS (%)	*p*
Concurrent chemotherapy							
Yes	29	83	0.927	86	0.724	76	0.851
No	11	82		82		73	
Pretreatment tumor diameter^a^							
< 40 mm	11	100	0.081	100	0.109	91	0.17
≥ 40 mm	29	76		79		69	
Pre-ICBT tumor diameter^a^							
< 40 mm	24	96	0.004	96	0.013	92	0.001
≥ 40 mm	15	60		67		47	
HR-CTV							
< 40 cm3	22	96	0.03	96	0.077	96	0.001
≥ 40 cm3	17	71		77		53	
Total HR-CTV D90							
≥ 70 Gy	29	90	0.044	93	0.018	83	0.044
< 70 Gy	11	64		64		55	
Overall treatment time							
< 57 days	28	93	0.009	93	0.035	89	0.001
≥ 57 days	12	58		67		42	

*PC* pelvic control, *LC* local control, *PFS* progression-free survival, *ICBT* intracavitary brachytherapy, *HR-CTV* high-risk clinical target volume

^a^Assessed by MRI T2WI

Prolonging OTT was associated with worse oncologic outcomes. Patients whose OTT was over 57 days had significantly more inferior oncologic outcomes compared with those whose OTT was less than 57 days.

### Complications

Table 6 shows late complications. The 2-year severe late complication (grade ≥ 3) rate was 3% for the bladder and rectum and 0% for the sigmoid colon and small bowel. The total bladder D2 cm^3^ of the 2 patients with grade 3 complications were 78 and 80 Gy. The patient who experienced grade 4 rectal complications had been receiving continuous anticoagulant medication for arrhythmia. Her total rectal D2 cm^3^ was 62 Gy. Concurrent chemotherapy had no significant influence on the incidence of complications.

**Table 6:** Numbers of patients with late complications

Organ	Grade 1	Grade 2	Grade 3	Grade 4
Bladder	2	2	2	0
Rectum	2	1	0	1
Sigmoid colon	0	0	0	0
Small bowel	2	0	0	0

Graded according to CTCAE v4.0

## Discussion

This single-institutional study of Japanese patients with cervical cancer treated with definitive RT/CCRT consisting of noCS-WPRT and CT-based ICBT demonstrated some important findings regarding feasibility, toxicity and oncologic outcomes. The results suggested several important issues to be solved in the future.

The planning aims for doses were successfully achieved for the rectum in most cases; only 3 patients could not attain the aims (noCS-WPRT + ICBT) of 66 Gy, and no patient exceeded the dose limit of 75 Gy recommended in the ABS guideline [26]. One patient developed G4 proctitis. The development of this adverse event may have been affected by the patient’s use of anti-coagulant medications, because the patient’s total D2 cm^3^ for the rectum was moderate (62 Gy). For the sigmoid colon and small bowel, some patients could not achieve the aims for single ICBT dose; consequently, 20 and 13%, respectively, did not accomplish the aims for total dose. However, all patients remained under the limit of 75 Gy recommended in the ABS guideline. As a result, no patient experienced severe (≥ G3) sigmoid and/or small bowel complications. In contrast, almost half of the ICBT sessions and cases could not achieve the aims for single and total doses for the bladder. One patient exceeded the total dose limit of 90 Gy recommended in the ABS guideline [26]. Consequently, two patients experienced G3 cystitis.

The 2-year LC rate of 85% in the present study seems poor compared with those of other 3D-IGBT series [27–30]. We suggest that one possible reason for the unfavorable result is insufficient total doses to the cervical tumor. The total HR-CTV D90 of 73 Gy in our series seems lower than those of the global series [20, 27–29, 31] and guidelines [1, 26]. Looking at LC by the total HR-CTV D90, the 2-year LC rate of the patients treated with 70 Gy and over was acceptable (93%) and significantly higher compared with that of those treated with less than 70 Gy (64%). From the findings, we hypothesize that a HR-CTV D90 of 70 Gy might be the minimum total dose required to achieve favorable LC in the most of patients.

For patients with a small HR-CTV (< 40 cm^3^) in this series, point A dose reduction was frequently indicated to remain within the dose constraints for the OARs. Regarding the protocol feasibility, compliance was poor for patients with a small tumor diameter assessed immediately before the 1st ICBT (pre-ICBT tumor diameter). This was probably due to the narrow distance between the ICBT applicators and the OARs. Despite such situations, most patients with a small pre-ICBT tumor diameter could receive an adequate total HR-CTV D90 of ≥ 70 Gy, and most achieved favorable LC. Based on the findings, we suggest that noCS-WPRT in combination with ICBT is applicable for patients with a small pre-ICBT tumor diameter.

We found that pre-ICBT tumor diameter had a negative impact on LC. We also found a negative correlation between HR-CTV and the D90 value. These results suggest that unfavorable LC for patients with a large pre-ICBT tumor diameter is due to the delivery of an insufficient HR-CTV D90. In fact, only 43% (7/16) patients with a HR-CTV ≥ 40 cm^3^ received ≥ 70 Gy of the total HR-CTV D90. The results suggest that escalation of the HR-CTV D90 is necessary to improve LC for patients with a large pre-ICBT tumor diameter with close monitoring of the OAR D2 cm^3^. In this series, there was little room remaining for the D2 cm^3^ until it reached the constraints for the OARs, especially the bladder, in patients whose HR-CTV was ≥ 40 cm^3^. Therefore, simple dose escalation seems difficult when considering the dose constraints for the OARs. Nishimura and coworkers reported the clinical significance of optimized treatment planning of MRI-based 3D-IGBT using tandem and ovoid applicators [12]. They demonstrated that it was difficult to increase the HR-CTV D90 while keeping within the D2 cm^3^ constraint of the OARs, especially the bladder, in patients with an extensive HR-CTV (≥ 40 cm^3^), despite the use of graphical optimization [12]. To overcome the limitation of dose distribution of standard ICBT, combined ICBT with interstitial brachytherapy (IC/IS brachytherapy) to a total HR-CTV D90 of 70 Gy could be a solution. In comparison with standard ICBT, IC/IS brachytherapy allows for escalation of the HR-CTV doses while keeping within the dose constraints of the OARs [32], and it achieves excellent results [33, 34].

This study has several limitations. First, a limited number of patients with stage III–IVA disease was included. Another multi-institutional prospective study of CCRT for patients with stage III–IVA disease was ongoing in the same time period, which might have affected the low accrual. Caution should be used when extrapolating the present results to patients with stage III–IVA disease. A second shortcoming of the study is the absence of HR-CTV contouring at the time of actual treatment planning. Although the process might have affected the feasibility of the protocol negatively, dose–response analyses could be applicable. Long OTT, with a median of 55 days in this series, is another serious flaw. Several studies have indicated that long OTT is one of the most important factors negatively affecting prognosis [31]. It resulted from an inappropriate treatment schedule planned in the protocol. To shorten the OTT, ICBT should be performed twice a week.

## Conclusion

The results of this study suggested that definitive RT/CCRT consisting of noCS-WPRT and CT-based ICBT may be feasible for Japanese patients with cervical cancer. The proper indication for IC/IS brachytherapy for cases with large pre-ICBT tumor diameter and optimized treatment schedules to shorten OTT are the next issues to further improve the oncologic outcomes without increasing toxicity.
