# Correction to: Definitive radiotherapy consisting of whole pelvic radiotherapy with no central shielding and CT-based intracavitary brachytherapy for cervical cancer: feasibility, toxicity, and oncologic outcomes in Japanese patients

**DOI:** 10.1007/s10147-023-02308-y

**Published:** 2023-02-16

**Authors:** Takeaki Kusada, Takafumi Toita, Takuro Ariga, Wataru Kudaka, Hitoshi Maemoto, Wataru Makino, Kazuki Ishikawa, Joichi Heianna, Yutaka Nagai, Yoichi Aoki, Sadayuki Murayama

**Affiliations:** 1 Department of Radiology, Graduate School of Medical Science, University of the Ryukyus, 207 Uehara, Nishihara, Okinawa 903-0215 Japan; 2 Radiation Therapy Center, Okinawa Chubu Hospital, 281 Miyazato, Uruma, Okinawa 904-2293 Japan; 3 Department of Obstetrics and Gynecology, Graduate School of Medical Science, University of the Ryukyus, 207 Uehara, Nishihara, Okinawa 903-0215 Japan; 4 Department of Obstetrics and Gynecology, Nanbu Medical Center/Nanbu Child Medical Center, 118-1 Arakawa, Shimajiri, Okinawa 901-1193 Japan

**Correction to: International Journal of Clinical Oncology (2020) 25:1977–1984** 10.1007/s10147-020-01736-4

In the original publication one of the funding numbers was incorrectly given as: JP19K08170. The correct number is: JP19K08179.

The updated Funding statement with correct number is given in this Correction as:

Funding This study was supported by the Japan Society for the Promotion of Science (JSPS) KAKENHI (Grant no. JP16K10398, JP19K08179).

